# Prophylactic treatment with oral azithromycin in cancer patients during the COVID-19 pandemic (OnCoVID): a randomized, single-blinded, placebo-controlled phase 2 trial

**DOI:** 10.1186/s13027-023-00487-x

**Published:** 2023-02-12

**Authors:** Maximilian J. Mair, Agnieszka Maj-Hes, Alina Nussbaumer-Pröll, Rainer Puhr, Agnieszka Christenheit, Marlene Troch, Hannah C. Puhr, Angelika M. Starzer, Ariane Steindl, Sabine Eberl, Helmuth Haslacher, Thomas Perkmann, Christoph Minichsdorfer, Gerald W. Prager, Wolfgang W. Lamm, Anna S. Berghoff, Barbara Kiesewetter, Markus Zeitlinger, Matthias Preusser, Markus Raderer

**Affiliations:** 1 Department of Medicine I, Division of Oncology, Medical University of Vienna, Waehringer Guertel 18-20, 1090 Vienna, Austria; 2 Department of Clinical Pharmacology, Medical University of Vienna, Vienna, Austria; 3 Department of Laboratory Medicine, Medical University of Vienna, Vienna, Austria

**Keywords:** COVID-19, SARS-CoV-2, Azithromycin, Prophylactic treatment, Oncology

## Abstract

**Background:**

Patients with cancer are at high risk for severe courses of COVID-19. Based on (pre-)clinical data suggesting a potential protective effect due to the immunomodulating properties of azithromycin, we have initiated a prospective randomized trial.

**Methods:**

This randomized, single-center, single-blinded, placebo-controlled phase 2 trial included adult patients with cancer undergoing systemic treatment. Patients were 1:1 randomized to oral azithromycin (1500 mg once weekly for 8 weeks) or placebo. The primary endpoint was the cumulative number of SARS-CoV-2 infections 12 weeks after treatment initiation.

**Results:**

In total, 523 patients were screened, 68 patients were randomized, and 63 patients received at least one dose of the study drug. Due to low acceptance and a lack of SARS-CoV-2 infections in the study cohort, the study was prematurely closed. With no reported grade III–IV possibly treatment-related adverse events, azithromycin was generally well tolerated. Overall survival (OS) rates after 12 months were 83.5% and 70.3% in the azithromycin and placebo group, respectively (*p* = 0.37). Non-SARS-CoV-2 infections occurred in 4/32 (12.5%) in the azithromycin and 3/31 (9.7%) in the placebo group (*p* = 1). No emergence of azithromycin-resistant *S. aureus* strains could be observed. According to treatment group, longitudinal alterations in systemic inflammatory parameters were detected for neutrophil/lymphocyte and leukocyte/lymphocyte ratios.

**Conclusion:**

Although efficacy could not be assessed due to premature closure and low incidence of SARS-CoV-2 infections, azithromycin was associated with a favorable side effect profile in patients with cancer. As other prophylactic treatments are limited, SARS-CoV-2 vaccination remains a high priority in oncological patients.

*ClinicalTrials.gov registration number and date (dd/mm/yyyy)*: NCT04369365, 30/04/2020.

**Supplementary Information:**

The online version contains supplementary material available at 10.1186/s13027-023-00487-x.

## Background

The SARS-CoV-2 pandemic has been declared a global public health emergency by the World Health Organization. In several studies, a significantly higher risk for severe disease and death has been observed in patients with cancer, especially those receiving ongoing systemic therapy [1, 2]. In the pandemic, several mRNA or viral vector vaccines have been developed which confer protection from SARS-CoV-2 infection and severe COVID-19 [3–5]. Although SARS-CoV-2 vaccination is generally recommended in patients with cancer, the efficacy of SARS-CoV-2 immunization was shown to be lower as compared to healthy individuals [6–11]. Therefore, further prophylactic treatment modalities protecting particularly vulnerable populations may be needed. Since the global spread of SARS-CoV-2, several drugs have been studied, including the antiviral drugs remdesivir and ritonavir, the antimalarial drug hydroxychloroquine, or the steroid dexamethasone, with varying degrees of efficacy [12–15]. Moreover, monoclonal antibodies including bamlanivimab/etesevimab, casirivimab/imdevimab, sotrovimab, and tixagevimab/cilgavimab have been approved for use as pre- and post-exposure prophylaxis (PEP) in patients at high risk for clinical deterioration due to SARS-CoV-2 infection [16–18]. However, the evidence for their use in patients with cancer is limited to retrospective analyses, and coverage of variants of concern (VOC), including the Omicron VOC, may be limited [19–21].

Azithromycin is a macrolide antibiotic targeting gram-positive, gram-negative, and atypical pathogens. Besides its antibacterial activity, immune-modulatory effects have been postulated, most probably due to a modulation of T cells, myeloid cells, and the release of inflammatory mediators such as interleukins (IL) 6/8 and tumor necrosis factor (TNF) alpha [22]. Thus, long-term azithromycin is increasingly and safely being used in other indications such as cystic fibrosis, bronchiolitis, and obstructive pulmonary diseases such as asthma [22]. One study in lung allograft recipients showed that prophylactic treatment with azithromycin was associated with decreased replication of human rhinovirus and lower levels of inflammatory cytokines [23]. Regarding SARS-CoV-2 infection, a small phase 2 trial evaluated a combination of hydroxychloroquine with azithromycin, with no detectable viral load after 6 days in 100% of patients receiving the combination as compared to 57.1% in the hydroxychloroquine only group [24].

Based on these findings, we designed this randomized, single-blinded, placebo-controlled phase 2 trial immediately at the beginning of the pandemic, when no vaccinations were yet available. The objective was to assess the efficacy and safety of oral azithromycin as a potential prophylactic treatment in patients with cancer receiving systemic antineoplastic therapy.

## Materials and methods

### Study design

The OnCoVID trial is a prospective, single-center, single-blinded, placebo-controlled randomized phase 2 trial performed at the Division of Oncology of the Medical University of Vienna to assess the efficacy of prophylactic azithromycin treatment in patients with cancer undergoing antineoplastic treatment during the COVID-19 pandemic. The full study protocol is given in Additional file 1.

In brief, eligible individuals were adult (≥ 18 years) patients with a histologically confirmed cancer diagnosis and a life expectancy of at least 3 months receiving systemic antineoplastic therapies regardless of the application route. Other inclusion criteria comprised an Eastern Cooperative Oncology Group (ECOG) performance status < 3 and a negative SARS-CoV-2 PCR at study inclusion as measured by routine real-time PCR testing for viral RNA. Ongoing or newly started adjuvant, neoadjuvant or palliative systemic anticancer therapies with either cytotoxic agents, molecular targeted therapies, monoclonal antibodies, checkpoint inhibitors, or combinations thereof were allowed. Exclusion criteria included the use of investigational agents within 28 days before study inclusion, patients with active opportunistic infections, ongoing radiotherapy, pregnancy or lactation, hypersensitivity to azithromycin or other macrolides, concurrent medication with ergotamine, theophylline, and digitalis, inadequate cardiac, hepatic, or renal function, a corrected QT interval (QTc) > 450 ms and the inability to swallow the study medication.

All study procedures were performed according to the Declaration of Helsinki with all applicable amendments. The trial was approved by the local ethics committee of the Medical University of Vienna (approval no. 1332/2020, 1164/2019) and was conducted according to local and institutional regulations. Informed consent was obtained from all participants before study inclusion. The trial is registered with clinicaltrials.gov (NCT04369365).

### Randomization and study procedures

Patients were 1:1 randomized to azithromycin monotherapy versus placebo. Blinded azithromycin or placebo was given orally at a dose of 1500 mg once weekly (3 × 500 mg pills) for a maximum of 8 consecutive weeks unless no SARS-CoV-2 infection was documented by a positive SARS-CoV-2 rt-PCR. Treatment was applied as two treatment cycles with 28 days each, with days 1, 8, 15, 22 constituting one treatment cycle. Randomization was performed with the Medical University of Vienna randomizer using block randomization stratified for sex and age (< 60 vs. ≥ 60 years).

Baseline assessments included documentation of the complete medical history including the oncological medical record, the oncological treatment plan and concomitant medication, physical examination and ECOG assessment, electrocardiogram (ECG) including QTc assessment, complete blood cell counts, routine serum biochemistry including procalcitonin and IL-6, and a pregnancy test in women with childbearing potential. Nasal swabs for the evaluation of resistant bacterial strains were obtained using Copan eSwab kits (Copan Diagnostics Inc., Murrieta, CA, USA) according to the manufacturer’s instructions. Regular study visits were performed on day one of each treatment cycle and at the end of the observation period. They included assessing adverse events, concomitant medication, physical examination, laboratory tests performed at baseline, ECG, and nasal swabs as described above. Real-time PCR for SARS-CoV-2 RNA was performed routinely prior to application of each cycle of antineoplastic treatment at our institution.

### Anti-SARS-CoV-2 antibody testing in patient serum

Blood samples of the included patients who provided written consent for biobanking of biological material were stored by the “MedUni Wien Biobank” facility according to Standard Operating Procedures in an ISO 9001:2015-certified environment [25]. Antibodies against the SARS-CoV-2 spike protein (anti-S) and the nucleocapsid (anti-NC) antibodies in patient serum were measured using Elecsys immunoassays (Roche Diagnostics, Rotkreuz, Switzerland) by the Department of Laboratory Medicine as published previously [6].

### Outcomes and endpoints

The primary endpoint was defined as the cumulative number of SARS-CoV-2 infections (both symptomatic and asymptomatic) detected by routine SARS-CoV-2 rt-PCR within 12 weeks after initiation of azithromycin or placebo. Secondary endpoints comprised the number of patients with severe COVID-19 (hospitalization of death) until 12 weeks after initiation of therapy, the severity of COVID-19 as classified in the WHO Blueprint for COVID-19 therapeutic trials [26], all-cause mortality, tolerance, and side effects related to azithromycin, rate of infections other than COVID-19 and the development of azithromycin-resistant bacterial strains. In exploratory post-hoc analyses, longitudinal alterations of systemic inflammatory parameters were evaluated.

### Assessment of azithromycin-resistant bacterial strains

ChromID *Staphylococcus aureus* (*S. aureus*) Elite agar (SAID, Biomérieux, Nürtingen, Germany), were used to cultivate samples from the nasal swabs and isolate *S. aureus* colonies. Columbia agar plates (+ 5% sheep blood, COS, Biomérieux) were used to grow overnight cultures of the reference strain and the clinical isolates for subsequent antibiotic susceptibility testing. Cation adjusted Mueller–Hinton–Broth (CAMHB, Sigma-Aldrich, Taufkirchen, Germany), containing 17.5 g/L casein acid hydrolysate, 3 g/L beef extract, and 1.5 g/L starch served as liquid media for minimal inhibitory concentration (MIC) testing. Azithromycin was obtained from Pfizer as 500 mg powder for infusion, then solved in 4.8 mL Aqua bidest., subsequently aliquoted in 0.5 mL and stored at − 80 °C until usage. The reference strain *S. aureus* ATCC-29213 from the American Type Culture Collection (ATCC) was used as comparator in the MIC testing (Azithromycin MIC range: 0.5–1 mg/L).

Swabs were processed within 5 days at room temperature (21 °C) or within 7 days stored in the fridge at 4 °C. Swabs were vortexed for 15 s, and 50 µL of the sample was dropped on the SAID plate. The sample was streaked out homogeneously on the plate with a sterile Q-tip to assure the best growing conditions for potential colonies. The plates were incubated at 37 °C for 24 h. Up to 3 pink colonies were isolated with a sterile Q-tip, put into a Cryo-tube with plastic beads, and stored at − 80 °C until used for MIC testing.

The reference strain *S. aureus* ATCC-29213 and clinical isolates of patients colonized with *S. aureus* throughout the whole study period were tested for their azithromycin susceptibility. Thus, for each bacterial strain, one plastic bead of the Cryo-tubes was placed on COS plates, a dilution streak was done, and they were incubated overnight at 37 °C. Colonies from the overnight culture were used to set a McFarland of 0.5 (~ 1.5 × 10^8^ cells) to inoculate 96-well plates with prior prepared azithromycin concentrations ranging from 32 to 0.06 mg/L in two-fold dilution steps.

The determination of the MIC for *S. aureus* ATCC-29213 and the clinical isolates was done in triplicates and according to the performance standards for antimicrobial susceptibility testing of the Clinical and Laboratory Standards Institute (CLSI) (National Committee for Clinical Laboratory Standards) in pure CAMHB.

### Statistical analysis

Initially, this study was designed as a phase 2 trial assuming infection rates of ~ 50% and an effect size of 15–20% difference in infection rates between groups, translating to a sample size of 200 patients to achieve a power of 56–81%. A predefined interim analysis was initially planned after 200 follow-up tests; however, closure of the trial due to futility was deemed possible in case of very low infection rates.

The intention to treat (ITT) population comprised all randomized patients. Subjects were analyzed according to their assigned treatment. Here, a modified ITT (mITT) set was defined where all patients who received at least one dose of the study drug were included. The per-protocol (PP) population included all patients who received the study drug without major protocol violations.

Analysis of the primary endpoint was performed by comparing the difference in SARS-CoV-2 infection rates between groups and by comparing time-to-event data using Kaplan–Meier plots and log-rank tests. Analysis of secondary endpoints are considered exploratory; therefore, no correction for multiple testing was applied [27]. Independence of categorical variables was tested using Chi-square or Fisher’s exact test as appropriate. Generalized linear mixed-effects models with normal distribution and log link were fitted to examine associations of oral azithromycin treatment with repeatedly assessed immunological marker levels. Random intercepts for participants and random slopes across visits with a heterogeneous unstructured covariance structure were allowed to account for individual variation in immunological markers. Effect modification was investigated by including an interaction term between the treatment indicator and the visit variables. All statistical analyses were conducted with R (version 4.1), and the significance level was set at a two-sided *p*-value of < 0.05.

## Results

### Patient characteristics

**Fig. 1:**
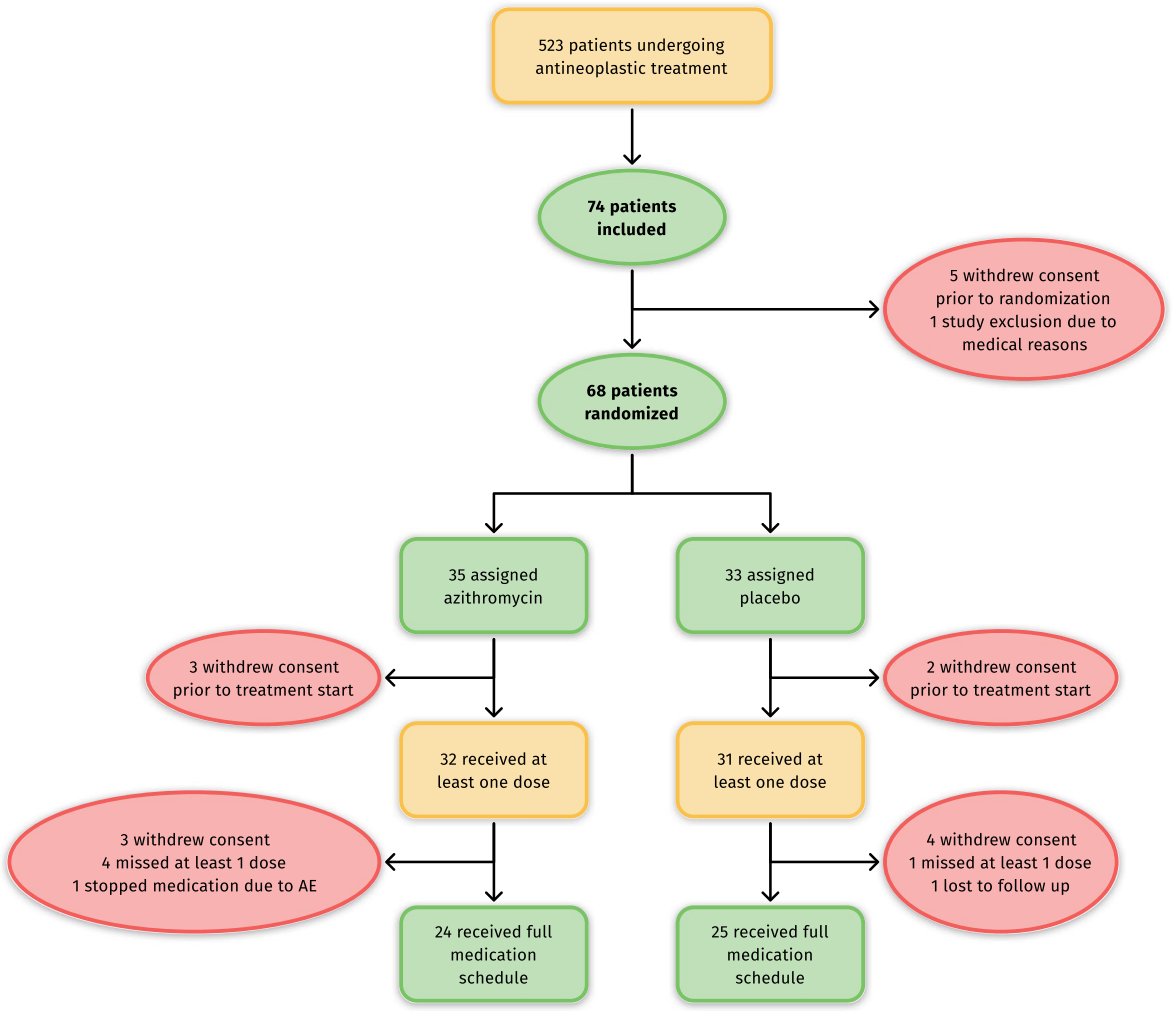
CONSORT diagram of the OnCoVID trial

Between April 27th, 2020 and April 30th, 2021, 523 patients undergoing antineoplastic treatment were screened, 74 were included, and 68 patients were randomized (CONSORT diagram in Fig. 1). Of those, 5 patients withdrew their consent before the first intake of the study medication. Consequently, 32 patients in the azithromycin and 31 in the placebo group received at least one dose of the medication (mITT population). The full study schedule was followed by 24 patients in the placebo and 25 patients in the azithromycin arms (PP population).

Median age was 59 years (range: 19–80) in the azithromycin and 62 years (range: 30–82) in the placebo group in the mITT population (*p* = 0.382, Mann–Whitney-U test). The most frequent tumor entities were colorectal cancer (7/32, 21.9%) in the azithromycin as well as lung cancer (6/31, 19.4%) and breast cancer (6/31, 19.4%) in the placebo group. In both treatment arms, most patients received chemotherapy as ongoing antineoplastic treatment. Further baseline characteristics are given in Table 1.

**Table 1:** Baseline characteristics in the modified intention to treat (mITT) population

	Azithromycin (n = 32)	Placebo (n = 31)	
Age at inclusion (median, range)	59 (19–80)	62 (30–82)	*p* = 0.382
*Gender*			
Male	20 (62.5%)	19 (61.3%)	*p* = 1.000
Female	12 (37.5%)	12 (38.7%)	
*ECOG at randomization*			
0	31 (96.9%)	28 (90.3%)	*p* = 0.355
1	1 (3.1%)	3 (9.7%)	
*Tumor entity*			
Colorectal cancer	7 (21.9%)	2 (6.5%)	*p* = 0.266
Lung cancer	6 (18.8%)	6 (19.4%)	
Pancreatic cancer	5 (15.6%)	5 (16.1%)	
Sarcoma	5 (15.6%)	2 (6.5%)	
Extranodal lymphoma	2 (6.3%)	2 (6.5%)	
Gastric cancer	1 (3.1%)	2 (6.5%)	
Cholangiocellular carcinoma	1 (3.1%)	1 (3.2%)	
Breast cancer	1 (3.1%)	6 (19.4%)	
Neuroendocrine carcinoma	0 (0.0%)	3 (9.7%)	
Other	4 (12.5%)	2 (6.5%)	
*Ongoing treatment*			
Chemotherapy	17 (53.1%)	17 (54.8%)	*p* = 0.539
Targeted therapy	2 (6.3%)	1 (3.2%)	
Immune checkpoint inhibition (ICI)	4 (12.5%)	3 (9.7%)	
Chemotherapy + targeted therapy	9 (28.1%)	7 (22.6%)	
Chemotherapy + ICI	0 (0.0%)	3 (9.7%)	

### Clinical study endpoints

Due to the low acceptance of the study and as no SARS-CoV-2 infections could be observed in the mITT and PP populations as detected by routine SARS-CoV-2 rt-PCR, the study was closed in April 2021. To assess undetected SARS-CoV-2 infections, we performed measurements of anti-SARS-CoV-2 nucleocapsid (anti-NC) and anti-spike (anti-S) antibody levels. In one patient, low levels of anti-NC antibodies could be measured at the end of the study; however, this individual was weekly tested negative by rt-PCR of respiratory specimen and showed no symptoms within the study period, suggesting a false positive anti-NC result in the absence of anti-S antibodies.

Azithromycin treatment was generally well-tolerated, as no grade III–IV possibly treatment-related adverse events were documented (Table 2). The most frequently reported adverse events (AEs) in the mITT population were diarrhea in 6/32 (18.8%) and abdominal cramps in 2/32 (6.3%) patients receiving azithromycin. Notably, one patient stopped intake of azithromycin due to diarrhea and abdominal cramps. In the placebo group, hypertension after intake of the study drug was documented in 2/31 (6.5%) patients, and syncope was observed in 1/31 (3.2%) patients. Clinically unsignificant QTc prolongation (grade I/II) was found in 7/32 (21.9%) patients in the azithromycin and 7/31 (22.6%) patients in the placebo group (*p* = 0.946, Chi-square test).

**Table 2:** Possibly treatment-related adverse events in the mITT population according to CTCAE v5

	Azithromycin (n = 32)	Placebo (n = 31)
Diarrhea (grade I–II)	6 (18.8%)	–
Abdominal cramps (grade I–II)	2 (6.3%)	–
Nausea (grade I–II)	1 (3.1%)	–
Fever (grade I–II)	1 (3.1%)	–
Dysgeusia (grade I–II)	1 (3.1%)	–
Syncope (grade III)	–	1 (3.2%)
Hypertension (grade I–II)	–	2 (6.5%)
QTc prolongation (grade I–II)	7 (21.9%)	7 (22.6%)

**Fig. 2:**
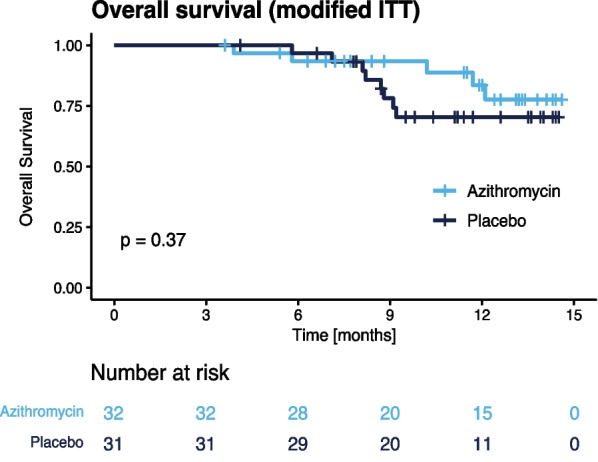
Overall survival of the mITT population in the azithromycin and placebo groups

All-cause mortality was comparable in both treatment arms (Fig. 2). Median overall survival (OS) was not reached. OS rates after 6 months were 93.4% (95%CI: 85.0–100%) and 96.7% (95%CI: 90.5–100%), while OS rates after 12 months reached 83.5% (95%CI: 69.7–100%) and 70.3% (95%CI: 54.9–90.0%) in the azithromycin and placebo groups, respectively (*p* = 0.37, log-rank test).

Other infections than SARS-CoV-2 were observed in 4/32 (12.5%) patients in the azithromycin and 3/31 (9.7%) patients in the placebo group, respectively (*p* = 1, Fisher’s exact test). These included venous catheter-associated infections (n = 2), urinary tract infections (n = 2), and febrile neutropenia, vaginal candidiasis, and positive blood cultures with unknown septic focus in one patient, each.

### Development of azithromycin-resistant Staphylococcus aureus strains

From 81 swabbed patients, 26 had at least at one visit a detected *S. aureus* colonization. This corresponds to ~ 32% and thus complies with the assumed *S. aureus* nasal colonization in humans [28].

For azithromycin susceptibility testing, only swabs from 9 patients were eligible, as they showed a *S. aureus* colonization at all study visits. Of these 9 patients, 5 were in the placebo group, and 4 were in the azithromycin group. Azithromycin-resistant *S. aureus* isolates were found in 4/9 (44.4%) patients and showed a MIC of over 32 mg/L during all visits. The resistant *S. aureus* isolates were isolated from one patient allocated to the placebo group and from 4 patients allocated to the azithromycin group. Clinical isolates from the 9 patients were either susceptible (0.5–1 mg/L) throughout the whole study period or were resistant from the beginning (> 32 mg/L). No emergence of resistance could be detected, neither in the placebo group nor in the azithromycin group. The individual MIC results are shown in Additional file 2: Table S1.

### Variations of systemic inflammatory markers over time according to treatment

In an exploratory post-hoc analysis, general linear mixed-effect models were fitted to assess whether systemic inflammatory markers were affected by azithromycin versus placebo treatment. The interaction term between treatment (azithromycin vs. placebo) and a visit was used to evaluate treatment-associated variations over time.

**Fig. 3:**
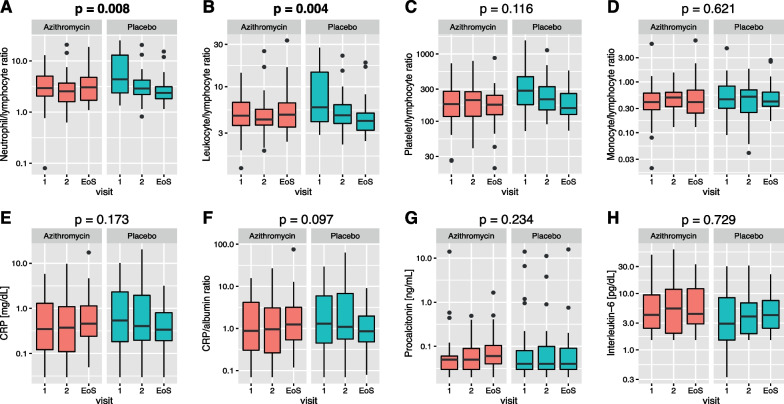
**A** Neutrophil/lymphocyte ratio, **B** leukocyte/lymphocyte ratio, **C** platelet/lymphocyte ratio, **D** monocyte/lymphocyte ratio, **E** C-reactive protein (CRP), **F** CRP/albumin ratio, **G** procalcitonin, and **H** interleukin-6 in the azithromycin and placebo groups at visit 1, 2, and end of study (EoS). *p*-values as determined by likelihood ratio test (LRT) for the interaction term between treatment group and visit

Significant associations were observed regarding the neutrophil/lymphocyte ratio (NLR; likelihood ratio test (LRT) for interaction terms: *p* = 0.008), with increasing NLR in the azithromycin and decreasing NLR in the placebo group (Fig. 3A). Similar results were seen for the leukocyte/lymphocyte ratio (LLR; LRT: *p* = 0.004, Fig. 3B). However, no significant alterations over time could be observed with regards to the platelet/lymphocyte ratio (PLR, Fig. 3C), the monocyte/lymphocyte ratio (MLR, Fig. 3D), C-reactive protein (CRP) levels (Fig. 3E), CRP/albumin ratio (Fig. 3F) as well as procalcitonin (PCT, Fig. 3G) and interleukin-6 (IL-6, Fig. 3H) levels. Descriptive statistics and details on fitted models are given in Additional file 2: Tables S2–S10.

## Discussion

To assess the prophylactic efficacy of azithromycin in patients with cancer, we performed this randomized, single-center, single-blinded, placebo-controlled phase 2 trial. Although the trial was designed and performed immediately at the beginning of the pandemic when no vaccines were available, the acceptance was low, with only 73/524 patients screened meeting the inclusion criteria and giving informed consent and 69 being randomized. In addition to the lower than expected recruitment, the primary endpoint could not be evaluated as there were no observed SARS-CoV-2 infections in the study cohort. Indeed, rigorous institutional policies and regular testing in patients and caregivers resulted in a lower rate of detectable SARS-CoV-2 infections at our department as compared to a control cohort without cancer in our hospital [29]. Consequently, pre-vaccination seroprevalence was low in patients and health care workers [30]. With the approval of SARS-CoV-2 vaccinations, patients with cancer were prioritized in vaccination campaigns due to their higher risk of contracting complications [31].

Given that antibody levels were lower in cancer patients than healthy controls, further measures may be needed to ensure maximum protection of patients with cancer and warrant the safety of continuing anticancer treatments during the pandemic [6–11]. In this regard, monoclonal antibodies inhibiting the binding of SARS-CoV-2 to human cells have been approved as pre- and post-exposure prophylaxis. However, future VOCs may further reduce the efficacy of the available monoclonal antibodies, underlining the need for additional prophylactic and therapeutic approaches in particularly vulnerable individuals.

Since the outbreak of the COVID-19 pandemic, a still growing number of studies assessing the efficacy and safety of repurposed drugs as prophylactic medication has been conducted. Hydroxychloroquine has been shown to decrease the viral load and persistence in an open-label non-randomized trial, especially if co-administered with azithromycin [24]. However, these results were intensively debated due to methodical shortcomings [32, 33], and other studies examining hydroxychloroquine only or in combination with other drugs were closed due to a lack of efficacy in interim analyses [34, 35]. The large, randomized PRINCIPLE trial compared azithromycin monotherapy with usual care ± other interventions in patients at high risk of an adverse clinical course of COVID-19. However, no significant differences in time to recovery and hospitalization rate between groups could be observed [36]. Patients receiving chemotherapy were included; however, specific subgroup analyses for patients with a compromised immune system were not reported. The CCC19 cohort study examined prognostic factors of patients with cancer afflicted from COVID-19 [37]. Interestingly, treatment with azithromycin and chloroquine was associated with increased 30-day all-cause mortality compared to those who received neither. In contrast, there was no significant difference in all-cause mortality for patients receiving azithromycin alone, although potential confounding for other indications could not be excluded. Overall, these data suggest a limited efficacy of azithromycin treatment to reduce the risk of severe COVID-19. In line, current guidelines do not recommend the use of azithromycin with or without hydroxychloroquine in COVID-19 in the absence of bacterial infections [21].

The safety of azithromycin in our trial was comparable to a previous study of prophylactic azithromycin treatment, where a serious adverse event was observed in only one out of 500 patients [36]. Data on tolerability of azithromycin in long-term regimens mainly stem from children with cystic fibrosis and bronchiectasis, confirming the excellent tolerability [38, 39]. Further supporting the favorable safety profile also for long-term administration in patients with cancer, only one grade 3 adverse event was documented in the MALT-A trial where azithromycin was applied once weekly for up to 6 months in patients with mucosa-associated lymphatic tissue (MALT) lymphoma [40]. Here, adverse events included mainly gastrointestinal symptoms such as nausea, diarrhea or abdominal pain. Moreover, serum and intracellular levels of azithromycin were observed to be relatively stable over time in once-weekly regimens, providing the pharmacokinetic basis for further long-term studies of azithromycin [41].

Among these lines, we could not observe the emergence of azithromycin-resistant bacterial strains. Yet, antibiotic resistance remains an issue, especially in the case of prophylactic administration. Indeed, although mass administration of azithromycin in children in sub-Saharan Africa has been shown to reduce mortality, a significantly higher prevalence of resistance gene determinants has been observed compared to children receiving a placebo even for non-macrolide antibiotics [42]. This is particularly important in patients with cancer due to their higher rates of resistance towards antibacterial, antifungal, and antiviral drugs and higher susceptibility for infections in general [43].

In our cohort, we could detect longitudinal alterations in systemic inflammatory parameters such as NLR and LLR according to the treatment group. Indeed, macrolides such as azithromycin display immune-modulatory properties such as a decreased release of cytokines and direct modulation of T cell and myeloid cell activity [22], potentially impacting systemic inflammation in patients with cancer. Macrolides are used in MALT lymphomas, where clinical and molecular evidence supports a direct antitumoral and immunomodulatory role besides the antibacterial activity against infectious agents such as *Helicobacter pylori* [44]. However, longitudinal changes of systemic inflammatory markers are influenced by a plethora of factors such as tumor progression, type of antitumoral treatment, and other inflammatory conditions [45, 46].

Our study has limitations. Due to the low number of included patients and no observed SARS-CoV-2 infections, the primary endpoint could not be evaluated, and the study was prematurely closed. Moreover, we enrolled all-comers undergoing antineoplastic treatment for solid tumors, inherently leading to heterogeneous cohorts in terms of disease entities and systemic antitumoral therapies. Further exploratory subgroup analyses and correction for confounding factors were therefore not feasible due to a small number of participants.

In conclusion, although the side effect profile of azithromycin was favorable, the efficacy of azithromycin in reducing SARS-CoV-2 infections and severe courses of COVID-19 could not be evaluated in our study. Given that other studies failed to show a benefit for azithromycin in COVID-19 and other prophylactic interventions remain scarce, SARS-CoV-2 vaccination remains pivotal to ensure the safety of vulnerable individuals such as patients with cancer.

## Supplementary Information

**Additional file 1**: Clinical trial protocol

**Additional file 2**: **Table S1**. Minimal Inhibitory Concentrations (MIC) of azithromycin resistance testing for individual patients in the placebo and azithromycin groups. **Table S2**. Descriptive data for inflammatory parameters of the included patients. **Tables S3–S10**. Generalized mixed model parameters for inflammatory parameters.

## Data Availability

Data may be shared upon reasonable request to the corresponding author.
