# Supplementary material for: Prophylactic treatment with oral azithromycin in cancer patients during the COVID-19 pandemic (OnCoVID): a randomized, single-blinded, placebo-controlled phase 2 trial
Source: Infect Agent Cancer. 2023 Feb 12;18:9. doi: 10.1186/s13027-023-00487-x (PMC9924847; doi:10.1186/s13027-023-00487-x)
Supplement: Supplementary file 1 — Additional file 1: Clinical trial protocol [file 13027_2023_487_MOESM1_ESM.pdf]

Clinical Study Protocol

A SINGLE-BLINDED RANDOMIZED, PLACEBO-CONTROLLED PHASE II TRIAL OF PROPHYLACTIC TREATMENT WITH ORAL AZITHROMYCIN VERSUS PLACEBO IN CANCER PATIENTS UNDERGOING ANTINEOPLASTIC TREATMENT DURING THE COVID-19 PANDEMIC

OnCoVID-19 Trial

Version 2.0/16.04.2020

EudraCT 2020-001327-13

CONFIDENTALITY STATEMENT

The information contained in this document is the property of the sponsor of this trial and therefore is provided to you in confidence for review by you, your staff, an applicable Ethics Committee/Institutional Review Board and regulatory authorities. It is understood that the information will not be disclosed to others without prior written approval from the sponsor, except to the extent necessary to obtain informed consent from those persons to whom the medication may be administered.

PROTOCOL INFORMATION

**Test drug (IMP)**	Azithromycin (oral)
**Protocol authors**	DDr. Barbara Kiesewetter-Wiederkehr<br>Ao Univ.-Prof. Dr. Markus Raderer
**Principal investigators**	Univ Prof Dr Matthias Preusser<br>Dept. of Internal Medicine I, Division of Oncology<br>Waehringer-Gürtel 18-20<br>1090 Vienna<br>e-mail: matthias.preusser@meduniwien.ac.at
**Document type**	Clinical study protocol
**Study phase**	Single blinded, placebo controlled randomized Phase II
**Version/Date**	2.0/216.04.2020

SPONSOR, INVESTIGATORS AND SIGNATURES

OnCoVID-19 Protocol, Version 2.0/16.04.2020

Sponsor: Medical University of Vienna

13.4.2020

Signature, Date

Univ.-Prof. Dr. Herbert Watzke
Department of Medicine I
Medical University of Vienna

I have thoroughly read and reviewed the study protocol "A single blinded, placebo-controlled randomized phase II trial of prophylactic treatment with oral azithromycin versus placebo in cancer patients undergoing chemotherapy during the COVID-19 pandemic" Version 2.0_ 16.04.2020. Having read and understood the requirements and conditions of the study protocol, I agree to perform the clinical study according to the international good clinical practice principles and regulatory authority requirements.

I understand that changes to the protocol must be made in form of an official amendment. I agree to report all serious adverse events, whether considered treatment-related or not within one working day.

16.4.2020

Signature, Date

Univ.-Prof. Dr Matthias Preusser
Department of Medicine I, Division of Oncology
Medical University of Vienna

COOPERATION PARTNERS

Ass.-Prof. Dr. Heimo Lagler
Department of Internal Medicine I
Division of Infectious Diseases and Tropical Medicine
Medical University of Vienna

Prof. Dr. Markus Zeitlinger
Department of Clinical Pharmacology
Medical University of Vienna

Prof Dr. Martin Posch and Dr. Franz König
Center for Medical Statistics, Informatics and Intelligent Systems,
Section of Medical Statistics
Medical University of Vienna

<u>Central Study Coordinators:</u>

Agnieszka Christenheit, MSc
Department of Oncology
Medical University Vienna

Ao. Univ Prof Dr Markus Raderer
Department of Oncology
Medical University Vienna

TABLE OF CONTENTS

1. PROTOCOL SYNOPSIS
2. BACKGROUND AND RATIONAL
    - 2.1 SARS-COV2 a novel respiratory virus causing a pandemic
    - 2.2 COVID-19 in cancer patients
    - 2.3 Azithromycin: background
    - 2.4 Rationales for performing the study
3. STUDY DESIGN AND OBJECTIVES
    - 3.1 Study design and endpoints
    - 3.2 Treatment groups and randomization
4. STUDY DURATION
5. NUMBER OF PATIENTS AND SELECTION CRITERIA
    - 5.1 Total number of patients
    - 5.2 Inclusion criteria
    - 5.3 Exclusion criteria
6. OUTCOME PARAMETERS
    - 6.1 Definition of primary endpoint
    - 6.2 Definition of secondary endpoints
    - 6.3 Definition of exploratory endpoints
7. STUDY MEDICATION
    - 7.1 Dosing of azithromycin
    - 7.2 Instructions for the use of azithromycin
    - 7.3 Packaging and Labelling
    - 7.4 Receipt of study drug
    - 7.5 Storage
    - 7.6 Unused drug supplies
8. STUDY PROCEDURES
    - 8.1 Screening and eligibility
    - 8.2 Study treatment
    - 8.3 Study procedures
    - 8.4 Dose continuation, modification and interruption
    - 8.5 Treatment compliance and drug accountability
    - 8.6 Concomitant Therapy

9. PREMATURE WITHDRAWAL
9.1 Premature trial termination
9.2 Replacement policy
10. STATISTICAL CONSIDERATIONS
10.1 Sample size calculation
10.2 Statistical analysis plan
10.3 Tolerability and safety
10.4 Statistical software
11. SAEFTY
11.1 Adverse events
11.2 Serious adverse events
11.3 Classification of severity
11.4 Classification of adverse events to IMP
11.5 Serious adverse event reporting
12. QUALITIY CONTROL AND QUALITY ASSURANCE
12.1 Periodic monitoring
12.2 Audits and inspections
12.3 Publication of study results
13. ETHICAL AND LEGAL ASPECTS
13.1 Informed consent of subjects
13.2 Acknowledgment and approval of the study
13.3 Protocol amendments
13.4 Study termination
13.5 Clinical study report
13.6 Finance and insurance
13.7 Ethics and good clinical practice
14. REFERENCES

1. PROTOCOL SYNOPSIS

TITLE	**A single blinded, placebo-controlled randomized phase II trial of prophylactic treatment with weekly oral azithromycin versus placebo in cancer patients undergoing antineoplastic therapy during the COVID-19 pandemic**
ACRONYM	OnCoVID-19
INDICATION	Prophylactic treatment in cancer patients undergoing antineoplastic therapy during the COVID-19 pandemic.
SCIENTIFIC RATIONAL	SARS-COV-2 is a novel corona virus causing a respiratory illness referred to as COVID-19, with corresponding symptoms ranging from mild respiratory tract infection to fatal acute respiratory distress syndromes and subsequent death. As of March 2020, more than 370,000 cases have been documented worldwide and the WHO has declared the current outbreak pandemic. To date there is no specific treatment for this highly contagious disease. Mortality rates are estimated at < 5%, but preliminary data suggest that cancer patients face a much higher risk of serious events and death, which can be explained by the compromised state of the immune system due to the malignancy itself and by anticancer treatments and chemotherapy in particular. The reported mortality rate for cancer patients is currently 40%. In addition, also the risk of SARS-COV-2 infection is thought to be increased in cancer patients due to these factors.<br><br>Oral azithromycin is a macrolide antibiotic commonly used for antibacterial treatment of respiratory tract infection. Side effects are rarely reported and consist mainly of mild nausea or dysgeusia. In addition to its antimicrobial effects, azithromycin inherits pronounced immunomodulatory properties including modulation of T-cells, NK-cells, dendritic cells and downstream cytokine release. These combined immunomodulatory and antibacterial effects are well established for the treatment of chronic lung diseases, with chronic obstructive pulmonary disease or cystic fibrosis representing important examples. Furthermore, also a potential antitumor activity of azithromycin was recently evaluated, and a weekly oral regimen was shown to be safe but only moderately active in the context of MALT lymphoma. Finally, in lung allograft recipients, azithromycin was able to reduce replication of rhinoviruses, suggesting potential effects also in the absence of bacterial infections.

	Current guidelines for treating severe COVID-19 cases, i.e. in patients in need of intensive care, recommend use of antiviral agents in addition to best supportive care, even though there is currently no definite evidence of efficacy of these agents and studies are yet ongoing. Application of broad spectrum antibiotics, e.g. azithromycin, is recommended if bacterial superinfection is suspected. Interestingly, preliminary data of a phase II trial evaluating the anti-malaria agent hydroxychloroquine against COVID-19 reported a superior efficacy of eliminating SARS-COV-2 if azithromycin is added, supporting the hypothesis that azithromycin is able to decrease viral replication in respiratory epithelia.<br><br>Given these data, we suggest that oral azithromycin can be safely and effectively used for prophylactic treatment of cancer patients undergoing antineoplastic treatment during the COVID-19 pandemic, with the aim to reduce both the risk of viral colonization of epithelium as well as to potentially reduce the risk of severe COVID-19 cases.
DESIGN / PHASE	Prospective, single center, single blinded placebo-controlled randomized, phase II trial
STUDY DURATION	Treatment will be given weekly for a maximum of 8 weeks in the absence of documented infection with SARS-COV-2.
CENTER	Medical University of Vienna, Department of Medicine I, Division of Oncology
COOPERATION PARTNERS	Medical University of Vienna, Department of Clinical Pharmacology<br><br>Medical University of Vienna, Department of Medicine I, Division of Infectious Diseases and Tropical Medicine<br><br>Medical University of Vienna, Center for Medical Statistics, Informatics and Intelligent Systems, Section for Medical Statistics
PATIENTS	The planned number of patients is 200 (100 per arm)
INCLUSION CRITERIA (SYNOPSIS)	- Histologically confirmed cancer diagnosis<br>- Ongoing systemic antineoplastic treatment irrespective of application route<br>- Age ≥ 18 years<br>- Life expectancy of at least 3 months<br>- Adequate renal, cardiac and liver function to tolerate the treatment

	- QTc ≤ 450 ms<br>- ECOG performance status of < 3<br>- Capable of understanding the study and giving informed consent<br>- Negative COVID-19 test at study entry as measured by routine testing (not older than 28 days)
EXCLUSION CRITERIA (SYNOPSIS)	- Use of any investigational agent within 28 days prior to study start<br>- Patients with active opportunistic infections<br>- Pregnant or lactating women<br>- Women of childbearing potential and male subjects not willing to use adequate contraception methods during the study period<br>- Hypersensitivity to azithromycin or other macrolides<br>- Concurrent medication with ergotamine, theophylline, digitalis<br>- Inability to swallow tablets
TEST DRUG (IMP) / TREATMENT	Azithromycin, applied as long-term treatment at once weekly dose of azithromycin 1500 mg (i.e. 3 x 500 mg on one day) orally.<br><br>Patients will be randomized to azithromycin monotherapy given orally once weekly at a dose of 1500 mg versus placebo. Treatment will last for a maximum of 8 weeks in the absence of documentation of SARS-COV-2-infection.
PRIMARY ENDPOINT	- Cumulative number of SARS-COV-2 infections (symptomatic or asymptomatic) as assessed by positive PCR from routine nasal swabs (performed every 28 days) at 12 weeks after initiation of therapy
SECONDARY ENDPOINTS	- Number of severe COVID-19 cases defined as combined endpoint of hospitalization rate or death at 12 weeks after initiation of therapy<br>- Severity of COVID-19 cases (see grading as outlined by the WHO)<br>- All-cause mortality<br>- Safety and tolerability of IMP (clinical and laboratory, CTCAE criteria)<br>- Number of viral and bacterial infections other than COVID-19<br>- Development of azithromycin-resistant bacterial strains
STUDY PROCEDURES	<u>At baseline (within 28 days prior to first treatment)</u><br><br>- Written informed consent<br>- Complete medical history including oncological medical record<br>- Assessment of the oncological treatment plan<br>- Documentation of concomitant medication

	- Physical examination and ECOG status assessment<br>- ECG including assessment of QTc<br>- Hematology (complete blood count, differential)<br>- Routine serum biochemistry including procalcitonin and IL6<br>- Pregnancy test in women of childbearing-potential<br><br><u>Investigations during the study and at the end of study visit</u><br><br>- Assessment of adverse events and concomitant medication<br>- Hematology (complete blood count, differential)<br>- Routine serum biochemistry including procalcitonin and IL6<br>- Pregnancy test in women of childbearing-potential<br>- ECG including assessment of QTc<br>- Nasal swabs for routine testing of potentially azithromycin resistant bacterial strains
STATISTICAL CONSIDERATIONS	<u>Efficacy</u><br><br>- Our hypothesis is that a reduction in the rate of PCR-evidenced infections as well as in hospitalization rates and/or death in infected patients at week 12 of at least 30 % could be considered clinically active.<br>- Following this, a total of 200 patients will be randomized 1:1 between azithromycin and oral placebo<br>- For this phase 2 study the sample size is chosen based on feasibility arguments. With a total sample size of 200 the two-sided confidence interval for the difference in proportions will at most extend 0.14 from the point estimate<br><br><u>Safety and tolerability</u><br><br>- Adverse events will be documented according to the CTCAE criteria as assessed by the investigator, results will be summarized using frequency counts and percentages<br><br><u>Final analysis</u><br><br>- All patients who are eligible for the study and receive at least one dose of study drug will be included in the analysis. Any patients who enroll into the study but who receive no study medication will be excluded, but a primary analysis will be conducted which will include all enrolled patients (intent to treat population)

2. BACKGROUND

2.1 SARS-COV2 a novel respiratory virus causing a pandemic

In December 2019, a first series of severe respiratory infections caused by a rapidly spreading virus was reported from China, and the responsible virus was defined as a novel enveloped RNA betacoronavirus termed *severe acute respiratory syndrome coronavirus 2 (SARS-COV-2)*, with the associated respiratory illness referred to as COVID-19 [1,2]. Based on the current knowledge, SARS-COV-2 is a highly contagious virus transmitted human-to-human via droplets from nose/mouth due to its high concentration in fluids of the respiratory tract, but the way of transmission is not yet fully understood and particularly aerosol and surface stability are currently under investigation[3]. In terms of clinical course, the symptoms are variable and range from asymptomatic cases to mild respiratory infections with cough, dyspnea and fever as leading symptoms, to pneumonia and severe acute respiratory distress syndrome (ARDS) with fatal consequences [4,5]. Particularly elderly patients and patients with (multiple) chronic diseases such as cardiovascular diseases and diabetes have been identified as main risk groups for fatal events and are at risk for early need of intensive care and respiratory support [6,7]. The estimated mortality rates range between <3% in the overall population to 10% in elderly patients and 40% in critically ill patients [8]. Therapeutic approaches for COVID-19 are currently mainly empirical, and rapidly developed guidelines recommend supportive care-only in mild cases while severe cases are treated arbitrarily with previously active antiviral compounds such as HIV protease inhibitors lopinarvir/ ritonavir, favipavir, remdesivir or anti-malaria drug hydroxychloroquine aiming to reduce the viral load [9-11]. However, while particularly the latter agent in combination with the oral macrolide antibiotic azithromycin has shown first positive data in a small series of 20 patients, there is currently no stringent evidence-based treatment recommendation [10].

As of March 2020, more than 370,000 cases and 16,000 deaths have been confirmed worldwide and the WHO has classified the situation as a pandemic outbreak and global health emergency situation with the focus of new cases currently transferred to Europe [12] . The rapidly increasing number of new infections results in a critical situation as many patients require intensive care and invasive ventilation which currently constitutes the main limiting factor in effectively caring for these patients. Thus, finding new therapeutic strategies and ways to limit spread of the disease but also mitigation measures to ensure a less aggressive clinical course in patients tested positive for COVID-19 are of utmost importance.

2.2 COVID-19 in cancer patients

Patients with an active diagnosis of cancer are prone to infections for multiple reasons, ranging from a potentially compromised immune system due the disease itself, tumor cachexia and malnutrition, to immunosuppression as the main side effect of most cancer treatments such as chemotherapy, molecular targeted agents and immunotherapy. A first analysis of a nation-wide cohort of 1590 COVID-19 patients in China identified 18 patients with a history of malignancy and sufficient medical data for detailed analysis [13], and reported an extrapolated three-fold higher rate of cancer patients within this collective if compared to the normal population (1% versus 0.29%). Furthermore, this analysis documented a significantly higher risk to develop severe symptoms, defined as the percentage of patients admitted to the intensive care unit requiring invasive ventilation or death, if compared to the overall population (39% vs 8%, p=0.0003). At particular high risk were patients with active treatment, with three of four patients undergoing antitumor therapy categorized as severe cases. Finally, the authors also reported a more rapid course of the disease with the median time to serious events being 13 versus 43 days (p<0.0001). While the numbers in this cohort are limited, these data clearly suggest cancer patients and particularly patients with ongoing treatment as a high risk group for severe COVID-19 manifestations. Whereas additional large cohorts on cancer patients are still lacking, this series identified age, lymphopenia and leucopenia but also concurrent treatment with prednisone as strong predictors of ARDS development and severe clinical course, with all of these factors being relevant in cancer patients [6,7]. In response to this, Onkopedia [14], a consensus platform for oncological care in German-speaking countries, has already prompted a specific guideline for managing cancer patients during the COVID-19 pandemic and suggested specific decision criteria for triaging application of anticancer treatments in view of infection risk and underlying cancer disease status.

2.2 Azithromycin: Background

Macrolide antibiotics are a class of antimicrobial compounds commonly used for a wide range of community acquired but also atypical infections targeting a broad spectrum of cocci bacteria, gram negative rods and atypical pneumonia pathogens. Beside their use as anti-bacterial agents, they have recently become a focus of interest in the context of “drug repurposing” in oncology and pulmonology due to pronounced immunomodulatory effects and direct antiproliferative features [15-17]. The suggested underlying mechanisms are mainly based on an increase in CD8+ cells, NK cells and interferon-gamma producing T-cells; furthermore macrolides decrease VEGF and TNF-alpha levels and act modulating on the release of

various cytokines including interleukins (IL) 6 and 8 [15,18]. Effects also include more specific mechanisms like alterations of the mTOR pathway [19]. In terms of clinical antitumor efficacy, clarithromycin showed activity in Waldenstrom's disease, multiple myeloma and more recently in mucosa-associated lymphoid tissue (MALT) lymphoma, a disease highly dependent on the tumor microenvironment and related to infectious diseases [15]. In two phase II trials and one large retrospective series, excellent tolerance and objective durable responses in 50% of patients were reported, and evaluation of the concept is ongoing [17,20,21]. Furthermore, azithromycin as long-term single agent once weekly (1500 mg) was evaluated for MALT lymphoma, and while the tumor response rate was inferior to clarithromycin at only 25%, tolerance and compliance were excellent, with no relevant adverse effects reported [22]. In spite of the fact that azithromycin appears to be less effective than clarithromycin in MALT lymphoma, the study showed stable drug-concentrations over the whole duration of therapy with excellent tolerance of 1500 mg (3 x 500 mg on a single day) weekly. The compound has also shown a broad range of immunomodulatory applications with established efficacy and clinical routine use in a variety of chronic lung diseases including obstructive pulmonary disease, cystic fibrosis, bronchiolitis obliterans syndrome or asthma [16,18].

In the current era of the COVID-19 pandemic, azithromycin is of therapeutic interest, and while it was initially only suggested as possible treatment for bacterial superinfection in severe COVID-19 cases, a highly featured article has reported that the antiviral effects of the anti-malaria compound hydroxychloroquine could be augmented by addition of azithromycin [10]. In these preliminary results of a small phase II trial, azithromycin was allowed as add-on to hydroxychloroquine 600 mg daily if clinically indicated (number of treated patients = 20). At day 6 post inclusion, no viral load was detectable in 70% of patients versus in 12.5% in the control group (p=0.001), and if results were further divided into patients receiving hydroxychloroquine alone or plus azithromycin, the rate of virological cure was 100% for the combination versus 57.1% for the hydroxychloroquine-only group. Differences in these outcomes were observed as early as from day 3 [10]. While the reasons for these effects of azithromycin are not fully understood and need to be further investigated, a further recent publication evaluating inflammation patterns in lung allograft recipients reported that prophylactic treatment with azithromycin was able to reduce the production of inflammatory cytokines but also to decrease the replication of human rhinovirus [23], suggesting substantial anti-viral effects due to pretreatment with this pleiotropic compound.

2.3 Rationales for performing the study

COVID-19 disease constitutes a global threat with a particular danger for immunocompromised individuals such as cancer patients undergoing active treatment with chemotherapy with an increased risk of infection and devastating mortality rates up to 40% on top of the already reduced life expectancy of cancer patients [13]. The macrolide antibiotic azithromycin has been shown to be effective for active and prophylactic treatment of respiratory infections and was additionally proven to not only act as an immunomodulator for the treatment of chronic lung diseases and but also appears to decrease viral replication in allograft lungs and potentially also in SARS-COV2 infected patients. Furthermore, azithromycin has a relatively good bioavailability and shows no significant side effects if applied long-term at a regular dose, e.g. 1500 mg per day once weekly as evidenced in the pilot trial of azithromycin for MALT lymphoma, while application of hydroxychloroquine may result in relatively severe toxicities. In view of these observations, we hypothesize that oral application of azithromycin 1500 mg orally once a week could effectively improve outcome of cancer patients undergoing antineoplastic treatment during the COVID-19 pandemic.

3. STUDY DESIGN AND OBJECTIVES

3.1 Study design and endpoints

Study design: This is a single-blinded, placebo-contolled , randomized, single center study to evaluate the capacity and safety of oral azithromycin as prophylactic treatment in cancer patients undergoing antineoplastic therapy including conventional chemotherapy +/- antibodies (bevacizumab, cetuximab, rituximab and obinotuzumab) or checkpoint-inhibitors. In this trial, patients will be randomized to azithromycin monotherapy orally once weekly at a dose of 1500 mg (3 x 500 mg taken on one day) versus standard of care with continued antineoplastic therapy only. Treatment will last for a maximum of 8 weeks in the absence of demonstrated infection with SARS-COV2.

Primary endpoints:

- Cumulative number of SARS-COV-2 infections as verified by PCR from routine nasal swabs performed every 28 days (symptomatic or asymptomatic) at week 12 after initiation of therapy

Secondary endpoints:

- To evaluate the capacity of azithromycin to prevent severe COVID-19 infections, defined as a combined endpoint of hospitalization and death in cancer patients undergoing active treatment with chemotherapy
- Severity of COVID-19 cases (see grading as defined by the WHO)
- All-cause mortality
- Safety and tolerability of IMP (clinical and laboratory, CTCAE criteria)
- Occurrence of viral or bacterial infections other than COVID-19
- Development of azithromycin-resistant bacterial strains as assessed by nasal swabs

3.2 Treatment groups and randomization

A 1:1 permuted block randomization with the MUW randomizer will be used. Eligible patients will be randomized 1:1 to either the treatment arm with the investigational agent azithromycin 1500 mg (3 x 500 mg) once weekly concomitantly with the start of antineoplastic therapy or 3 tablets of oral placebo as prepared and labelled by the local pharmacy. Randomization should occur as close as possible to treatment start (i.e. optimal within 24 hours, maximum 3 days prior to treatment start). Patients will be randomized with the online randomization program "Randomizer" (https://www.meduniwien.ac.at/randomizer/web/login.php). Registered persons only can perform randomization. For login, an individual User-ID and password entry are necessary. Hence, randomization can only be conducted by the Central Study Coordinators and/or the PI.

Randomization will be conducted only if the patient can be included in the study (checking of inclusion- and exclusion criteria, written consent of the patient). The advantage of the online randomization with the "Randomizer" is that for each patient, information will be available, when the patient was randomized and by whom. After successful randomization, the EVLA will start within 30 minutes.

A block randomization will be used, stratified for sex and age (< 60 vs. >= 60). Patients will be randomized to one of the following two groups:

- Arm A: continued oncological therapy plus azithromycin (Zithromax) 1500 mg orally (3 x 500 mg tablets) once a week for 8 weeks in the absence of documented SASR-COV2 infection or adverse events
- Arm B: continued oncological therapy plus placebo 3 tablets daily orally once a week for 8 weeks in the absence of documented SASR-COV2 infection or adverse events

4. STUDY DURATION

Duration of the study: 5 months

Recruitment period: 2 months

First patient in: April 25, 2020

Last patient out: August 25, 2020

5. NUMBER OF PATIENTS AND SELECTION CRITERIA

5.1 Total number of patients

A total number of 200 patients (100 patients in each arm) is planned for enrollment in this pilot trial. For statistical considerations and sample size calculation see section on Statistics.

5.2 Inclusion criteria

To be included each patient must fulfill all of the following criteria:

- Histologically confirmed cancer diagnosis
- Active disease in need of antineoplastic therapy
    - treatment may include classical cytostatic agents, molecular targeted therapy, monoclonal antibodies bevacizumab, cetuximab, rituximab and obinutuzumab (either alone or in combination with chemotherapy) and checkpoint inhibitors
    - treatment may be ongoing or newly started
    - oral, intravenous or subcutaneous application route is allowed

    - adjuvant, neoadjuvant or palliative chemotherapy is allowed
    - the planned treatment duration must be at least 3 months, i.e. 12 weeks
- Age ≥ 18 years
- Life expectancy of at least 3 months
- Adequate renal, cardiac and liver function to tolerate the treatment
- Normal QTc (≤ 450 ms) on ECG
- ECOG performance status of < 3
- Negative PCR for SARS-COV-2 not older than 28 days
- Capable of understanding the study and giving informed consent

5.3 Exclusion criteria

<u>Patients who fulfill any of the following criteria will be excluded:</u>

- Active COVID-19 as defined by clinical WHO criteria and a positive PCR for SARS-COV2
- Use of any investigational agent within 28 days prior to study start
- Ongoing radiotherapy
- A history of uncontrolled seizures, central nervous system disorders or psychiatric disability judged by the investigator to be clinically significant and adversely affecting compliance to study drugs
- Clinically significant cardiac disease including unstable angina, acute myocardial infarction within six months prior to randomization, congestive heart failure (NYHA III-IV), arrhythmia unless controlled by therapy, with the exception of extra systoles or minor conduction abnormalities,
- long QT syndrome (QTc interval >450ms).
- Subjects who have current active hepatic or biliary disease (with exception of patients with Gilbert's syndrome, asymptomatic gallstones, liver metastases or stable chronic liver disease per investigator assessment)
- Inadequate kidney function: serum-creatinine >2.0 times upper normal limit
- Hepatic dysfunction: total bilirubin >1.5 times upper normal limit (unless due cancer involvement of liver or a known history of Gilbert's disease); ALT >2.5 times upper normal limit (unless due to disease involvement of liver); alkaline phosphatase >2.5 times upper normal limit (unless due to disease involvement of the liver or bone marrow)
- Patients with active opportunistic infections
- Pregnant or lactating women. Women of childbearing potential must have a negative pregnancy test at screening, pregnancy testing must be performed within 7 days of administration of IMP. Approved methods of birth control must be used

- Women of childbearing potential, including women whose last menstrual period was less than one year prior to screening, unable or unwilling to use adequate contraception from study start to one year after the last dose of protocol therapy. Adequate contraception is defined as hormonal birth control, intrauterine device, double barrier method or total abstinence.
- Male subjects unable or unwilling to use adequate contraception methods from study start to one year after the last dose of protocol therapy
- Hypersensitivity to azithromycin or other macrolides
- Concurrent medication with ergotamine, theophylline or digitalis
- Inability to swallow tablets/study medication

6. OUTCOME PARAMETERS

6.1 Definition of the primary endpoint

Number of SARS-COV-2 infections (symptomatic or asymptomatic): detected by routine SARS-COV-2 tests in all patients treated at the Division of Oncology, Medical University of Vienna. During the SARS-COV-2 pandemic, the routine approach to patients undergoing chemotherapy at our institution is PCR from nasal swabs taken every 28 days. The cumulative number of infections detected (symptomatic or asymptomatic) at week 8 after initiation of therapy in both treatment arms serves as the primary endpoint.

6.2 Definition of secondary endpoints

Number of patients experiencing severe COVID-19 infection defined as a combined endpoint of hospitalization or death at week 12 after initiation of therapy.

COVID-19 cases are defined as outlined by the WHO, including a PCR for COVID-19 from any specimen (respiratory, blood, urine, stool, other bodily fluids).

Severity of COVID-19 cases: The severity of cases will be classified as suggested in the Blue Print for COVID-19 therapeutic trials by the WHO (range 0-8)

Table. Ordinal Scale for Clinical Improvement of COVID-19 (WHO Blueprint).

Patient state	Descriptor	Score
Uninfected	No clinical or virologicaly evidence of infection	0
Ambulatory	No limitations of activity<br>Limitation of activities	1<br>2
Hospitalized – mild disease	Hospitalized, no oxygen therapy<br>Oxygen by mask or nasal prongs	3<br>4
Hospitalized – severe disease	Non-invasive ventilation or high flow oxygen<br>Intubation and mechanical ventilation<br>Ventilation + additional organ support – pressors, RRT, ECM	5<br>6<br>7
Dead	Death	8

All cause mortality

Tolerance and side effects related to azithromycin

Rate of infections other than COVID-19, i.e. bacterial and viral infections

Development of azithromycin-resistant bacterial strains:

- During our study, the influence of the macrolide on resistance of S. aureus strains in the nasal flora vs placebo will be assessed by nasal swabs (taken at basline and then at Visit 1 + 2, i.e. week 4 and 8 latest). S. aureus strains will be cultivated on selective agar-plates, then isolated and frozen in Cryo-tubes for resistance testing at a later time-point.
- *S. aureus* will be cultivated and isolated on Chrom-ID Elite Plates:
- https://www.biomerieux.de/klinische-diagnostik/chromidtm-s-aureus-elite-agar-saide
- Testing for resitance will be done phenotypically using a micro-dilution method.

7. STUDY MEDICATION

7.1 Dosing of azithromycin

Azithromycin (Zithromax) will be made available as labelled study drug (label: azithromycin/placebo 500 mg) by the Sponsor and will be stored at room temperature (not above 30 °C). Dosage form: white, capsule-shaped, film-coated 500 mg tablet, packed in one blister for 3 tablets.

Placebo (film-coated maltodextrin): will be made available by the local pharmacy and labelled as azithromycin/placebo 500 mg packed in one blister for 3 tablets.

7.2 Instructions for the use of azithromycin(placebo

One weekly dose of azithromycin 1500 mg orally will be applied (3 x 500 mg pills of azithromycin weekly, taken on a single day), with days 1, 8, 15 and 22 constituting one treatment cycle. Two cycles given every 28 days are planned. It is not required to administer any pre-medication for treatments with azithromycin. Placebo will be be applied in a similar way, i.e. 3 tablets daily once a week days 1, 8, 15 and 22 for two cycles every 28 days (total of 8 weeks)

7.3 Packaging and labelling

The sponsor is responsible to provide the shipping orders. Azithromycin/Placebo will be provided and labelled by the sponsor of this trial. The drugs will be provided to the sponsor in individual pharmaceutical packings. Study medication must be dispensed in the original packaging with the label clearly visible. Each pharmaceutical packing will identify the contents as study medication and bear protocol number. In addition, the label will bear the sponsors name, quantity contained, a package number and the standard caution statement as follows: Caution: New drug - Limited by Federal law to investigational use. The study drug label must be clearly visible.

7.4 Receipt of the study drug

The investigator or designee is responsible for taking an inventory of each shipment of study drug received and comparing it with the accompanying study drug accountability form. The investigator will verify the accuracy of the information on the form, sign and date it, retain a copy in the study file. The numbers of pharmaceutical packings must be recorded when drug is received and dispensed.

7.5 Storage

At the study site, all investigational study drugs for oral use will be stored in a locked, safe area to prevent unauthorized access. The study drug should not be stored above 30 °C.

7.6 Unused supplies

Returned or unused study drug will be transferred to the hospital pharmacy and destroyed due to local SOP´s. If any study drug is lost or damaged, its disposition should be documented in the source documents and Drug Dispensing LOG.

8.STUDY PROCEDURES

8.1 Screening and eligibility

The Investigator is responsible for keeping a record of all subjects who sign an informed consent form (ICF) for entry into the study. All subjects will be screened for eligibility. Investigations at baseline and during the study are outlined below. Study Assessments, unless otherwise specified, must take place within 28 days prior to initiation of therapy. Screening pregnancy tests for women of childbearing potential must occur within 7 days before first application of investigational medical product (IMP). Before screening, a negative SARS-COV-2 PCR not older than 28 days must be present.

8.2 Study treatment

Patients will be randomized in 1:1 ratio in Arm A or B:

Arm A: continued oncological therapy plus azithromycin (Zithromax) 1500 mg orally once a week for 8 weeks in the absence of documented SASR-COV2 infection or adverse events
Arm B: continued oncological therapy plus placebo 3 tablets orally daily once a week for 8 weeks in the absence of documented SASR-COV2 infection or adverse events

8.3 Study procedures

At baseline (within 28 days prior to first treatment)

- Written informed consent
- Complete medical history including oncological medical record
- Assessment of the oncological treatment plan
- Documentation of concomitant medication

- Physical examination and ECOG status assessment
- ECG including assessment of QTc
- Hematology (complete blood count, differential)
- Routine serum biochemistry including procalcitonin and IL6
- Pregnancy test in women of childbearing-potential
- Nasal swab for resistance testing at baseline

<u>Investigations during the study (study visits on day one of each antineoplastic treatment cycle or on day 28 latest) and at end of treatment</u>

- Assessment of adverse events and concomitant medication
- Hematology (complete blood count, differential)
- Routine serum biochemistry including procalcitonin and IL6
- Pregnancy test in women of childbearing-potential
- ECG with assessment of QTc
- Nasal swab for testing of azithromycin-resistant bacterial strains at Visit 1 and 2 (latest week 4 and 8)

PCR for SARS-COV-2 from nasal swabs taken every 28 days is part of the routine care of patients; however, results will be used for defining infection with SARS-COV-2.

8.4 Dose continuation, modification and interruption

No dose modifications are planned. Doses can be withheld at the discretion of the physician for a maximum of 28 days. In case of PCR-positivity for SARS-COV-2 at any time during the 8 or placebo week study period, therapy with azithromycin will be immediately discontinued.

8.5 Treatment compliance and drug accountability

At all times, study drug will be prepared and administered by study site personnel, and patients will be given study for one treatment cycle (i.e. 4 weekly doses) every 28 days. Patients are asked to bring any left-over medication back to the study site. Quantity and LOT-numbers of used vials/pharmaceutical packings must be recorded on “Drug Dispensing LOG´s” and in the source documents.

8.6 Concomitant therapy

Subjects should receive full standard of care, including transfusions of blood and blood products and antiemetics when appropriate. No specific concomitant medication is prohibited, but all compounds have to be documented appropriately.

9. PREMATURE WITHDRAWAL

9.1 Premature trial termination

Any patients who experience a serious adverse event may be withdrawn at any time from the study at the discretion of the investigator. If any serious disadvantage of the treatment becomes evident during the clinical trial, therapy will be terminated. In this case the necessary procedures will be arranged to ensure protection of the subjects' interests.

9.2 Replacement policy

A total of N=200 patients, i.e. 100 per treatment group, will be enrolled into the study; If patients are withdrawn for reasons other than SARS-COV2 infection within the first 4 weeks of the study (i.e. before the first response evaluation), they will be replaced.

10. STATISTICAL CONSIDERATIONS

10.1 Sample size calculations

Reliable sample size planning is challenging because of the unpredictability and dynamics of the incidence rates in this epidemy. However, upper bounds for the accuracy of confidence bounds and lower bounds for the power of hypothesis tests can be given. For the sample size calculation, asymptotic z-tests and chi-squared tests were used (the actual analysis will account for time trends in the incidence rates).

For this **phase 2 study** the sample size is chosen based on feasibility arguments. With **a total sample size of 200** the two-sided confidence interval for the difference in proportions will at most extend 0.14 from the point estimate (computed with the large sample normal distribution). If the null hypothesis of equality of proportions is tested with a 2-sided chi-squared test at level 0.05, the power is at least 81% if the true treatment effect is 20 percentage points (e.g., incidence rates of 30% in the treatment and 50% in the control group). If the difference is 15 percentage points the power is at least 56%.

Distance of two-sided 95%-confidence bound to the observed value for the difference in proportions (large sample approximation)

Sample Size (total)	100	**200**	400	600	800
Distance of CI from observed proportion	±0.2	**±0.14**	±0.1	±0.08	±0.07

Power to for the chi-squared test the test the null hypothesis of equal proportions (2-sided significance level 0.05). The power below is calculated assuming the infection rate are symmetrical around 50%, e.g., assuming an effect size of 10 % points would translate to infection rates of 45% and 55%, respectively.

**Effect Size (Percentage Points)**	**Sample Size (total)**				
	100	**200**	300	400	800
7	10%	**17%**	23%	29%	51%
10	17%	**29%**	41%	52%	80%
15	32%	**56%**	74%	85%	99%
20	52%	**81%**	93%	98%	99%

Recruitment period: Recruitment period 4 Weeks (50 patients per week), follow-up 12 weeks After 200 follow-up tests have been performed (around week 8), an interim and safety analysis will be performed. If very low infection rates, i.e. less than one infection, are observed the trial may be stopped for futility. If the results are promising, the trial might be extended (further details see in section on statistical analysis).

10.2 Statistical Analysis Plan

Analysis Sets

Two different analysis sets are defined

Modified Intention to treat set (mITTs)

This analysis set includes subjects who were randomized. According to the intent to treat principle, subjects will be analyzed according to the treatment they have been assigned to during the randomization procedure. The miTT includes all patients who are eligible for the study and receive at least one dose of study drug will be included in the analysis. Any patients who enroll into the study but who receive no study medication will be excluded, but a primary analysis will be conducted which will include all enrolled patients (intent to treat population)

Per-protocol set (PPs)

This analysis set comprises all subjects who received study drug (at least one dose) and did not violate the protocol in a way that might affect the evaluation of the effect of the study drug(s) on the primary objective, i.e., without major protocol violations.

Baseline parameters and concomitant medications

Baseline parameters, medical history and concomitant medication will be documented during screening and throughout the trial until week X. Enrolment, protocol deviations and discontinuations from the study drug and the study will be summarized. Demographics (age, race, ethnicity and sex) and medical history and study drug administration will also be summarized by treatment group. For qualitative variables (e.g. sex), absolute (n =) and relative frequencies will be calculated per treatment group. Data will be visualized by bar plots. For quantitative data (e.g. age), the number of valid observations (n =), mean, standard deviation, standard error, median, minimum and maximum will be calculated for each treatment group and each time point separately. Data will be visualized by boxplots and histograms.

Primary Endpoint Analysis

All primary analyses will be on the ITT set and the analysis based on the per protocol set will be provided as supportive analysis. The primary endpoint is the between group difference in the incidence rate per 4 weeks. We will compute 2-sided 95% confidence bounds for the incidence rates. Furthermore, we test the null hypothesis that the incidence rates in the two groups are equal. To estimate and compare the incidence rates, the test status at weeks 4,8,12 will be modeled using time to event analysis models and will adjusted for calendar time to account for time trends in the incidence rate. The time-to-infection is defined as time from randomization to infection. If patients drop out, their observations will be censored at the last observation time.

This data will be visualized by Kaplan-Meier Plots. For each group unadjusted two-sided 95% confidence intervals will be reported as well.

Secondary Endpoints

- Number of severe COVID-19 cases defined as combined endpoint of hospitalization rate or death at 12 weeks after initiation of therapy
- Severity of COVID-19 cases (see grading by WHO)
- All-cause mortality
- Safety and tolerability of IMP (clinical and laboratory, CTCAE criteria)
- Infections other than COVID-19 including bacterial and viral infections
- Occurrence of azithromycin-resistant bacterial strains

For **binary secondary endpoints** (such all-cause mortality or number of severe COVID-19 cases) absolute (n =) and frequencies in percent (%) will be calculated per treatment group. 95%-confidence intervals will be calculated for rates, if appropriate. Such data will be visualized with bar charts. If appropriate, logistic regression models will be applied using treatment as independent factor. Furthermore, the model will be adjusted for sex and age.

For **ordinally scaled endpoints** (such as Grading of COVID-19 cases on day 0, 28 (=week 4), 56 (=week 8) and 84 (=week 12) after randomization) absolute (n=) and frequencies in percent (%) will be calculated for all categories separately for each treatment group (and timepoints. If appropriate, treatment groups will be compared with each other by Mann-Whitney-U Tests.

For **time-to-event endpoints** (such as overall survival (OS)) Kaplan-Meier plots will be provided. The two-group will be compared with log-rank Tests. Additionally, similar analysis as for the primary endpoint will be performed.

The analyses of all secondary endpoints are considered as exploratory. Unadjusted p-values and 95%-confidence intervals might be presented for secondary endpoints, but these are for descriptive purposes only and not subject to adjustment for multiplicity.

Interim analysis and extension of the study

After 200 follow-up tests have been performed (around week 8) an interim and safety analysis will be performed. If very low infection rates are observed the trial may be stopped for futility.

The phase II study might be extended to a confirmatory study in case the results of the pilot study are promising. Then this phase II pilot study will become the first stage of an adaptive design and the stagewise results will combined via stagewise p-values using the inverse normal combination function [24,25]. The weight for the first stage in the inverse normal combination function will be fixed to w1=Sqrt(0.5) and the p-value of the first stage will be based on the time-to-event analysis defined for the primary endpoint above [26]. The sample size for the second part will be determined by using data provided by the phase II trial. Depending on the sample size then needed, the study might be extended to other centers. In any case before the study is extended, a major protocol amendment will be performed and submitted to the relevant ethics committee(s). Further details on the statistical methodology will be provided in the statistical analysis plan (SAP, which will be finalized before the first interim analysis.

10.3 Tolerability and safety

Safety and tolerance of treatment in terms of hematologic and non-hematologic side effects as assessed by the investigators according to the most recent version of the CTCAE criteria (V5.0). Adverse events will also be summarized using frequency counts and percentages.

10.4 Statistical Software

All statistical analyses will be conducted with statistical software like SAS 9.4. (or higher) and R 3.6.3. (or higher).

11. SAFETY

11.1 Adverse events

An adverse event (AE) is any noxious, unintended, or untoward medical occurrence occurring at any dose that may appear or worsen in a subject during the course of a study. It may be a new intercurrent illness, a worsening concomitant illness, an injury, or any concomitant impairment of the subject’s health, including laboratory test values (as specified by the criteria

below), regardless of etiology. Any medical condition that was present prior to study treatment and that remains unchanged or improved should not be recorded as an AE. If there is a worsening of that medical condition this should be considered an AE. A diagnosis or syndrome should be recorded on the AE page of the Case Report Form rather than the individual signs or symptoms of the diagnosis or syndrome. All AEs will be recorded by the investigator from the time of signing the informed consent through to the end of the designated follow-up period.

11.2 Serious adverse events

A serious adverse event (SAE) is any AE which:

- Results in death
- Is life-threatening (i.e., in the opinion of the Investigator(s) the subject is at immediate risk of death from the AE)
- Requires inpatient hospitalization or prolongation of existing hospitalization
- Results in persistent or significant disability/incapacity (a substantial disruption of the subject's ability to conduct normal life functions)
- Is a congenital anomaly/birth defect
- Constitutes an important medical event

Important medical events are defined as those occurrences that may not be immediately life threatening or result in death, hospitalization, or disability, but may jeopardize the subject or require medical or surgical intervention to prevent one of the other outcomes listed above. Medical and scientific judgment should be exercised in deciding whether such an AE should be considered serious. Events not considered to be SAEs are hospitalizations which: were planned before entry into the clinical study; are for elective treatment of a condition unrelated to the studied indication or its treatment; occur on an emergency outpatient basis and do not result in admission (unless fulfilling other criteria above); are part of the normal treatment or monitoring of the studied indication and are not associated with any deterioration in condition. If an AE is considered serious, both the AE pages of the CRF and the SAE Report Form must be completed. For each SAE, the Investigator will provide information on severity, start and stop dates, relationship to study drug, action taken regarding study drug, and outcome.

11.3 Classification of severity

For both AEs and SAEs, the investigator must assess the severity of the event. The severity of adverse events (AEs) will be graded on a scale of 1 to 5 according to the National Cancer Institute (NCI) Common Terminology Criteria for Adverse Events Version 5.0 (NCI CTCAE).

If a specific event is not included in the NCI CTCAE toxicity scale, the following scale should be used to grade the event

Table. General NCI CTCAE adverse event grading scale.

Grade 1	**Mild** - Awareness of sign, symptom, or event, usually transient, requiring no special treatment and generally not interfering with usual daily activities
Grade 2	**Moderate** - Discomfort that causes interference with usual activities; usually ameliorated by basic therapeutic maneuvers
Grade 3	**Severe** - Incapacitating with inability to do usual activities or significantly affects clinical status and warrants intervention. Hospitalization may or may not be required
Grade 4	**Life-threatening** - Immediate risk of death; requires hospitalization and clinical intervention
Grade 5	**Death**

11.4 Classification of adverse events to IMP

The investigator must determine the relationship between the administration of study drug and the occurrence of an AE /SAE as “not suspected” or “suspected” to be related to IMP as defined below:

Not suspected: The temporal relationship of the adverse event to study drug administration makes a causal relationship unlikely or remote, or other medications, therapeutic interventions, or underlying conditions provide a sufficient explanation for the observed event.

Suspected: The temporal relationship of the adverse event to study drug administration makes a causal relationship possible, and other medications, therapeutic interventions, or underlying conditions do not provide a sufficient explanation for the observed event.

11.5 Serious adverse event reporting

Reporting to the regulatory authorities and ethics committee

The sponsor will inform relevant Regulatory Authorities and the Ethics Committee of all relevant information about serious unexpected adverse events suspected to be related to the study medication (SUSARs) that are fatal or life threatening as soon as possible, and in any

case no later than seven days after knowledge of such a case. Relevant follow-up information for these cases will subsequently be submitted within an additional eight days and of all other serious unexpected events suspected to be related to the study medication (SUSARs) as soon as possible, but within a maximum of fifteen days of first knowledge by the investigator.

Immediate reporting: The investigator will inform the sponsor of all SAEs within 24 hours in order that the sponsor can fulfil his regulatory reporting obligations within the required timeframes. Summary of product characteristics (SmPC) will be provided by the sponsor in the currently available form.

Contact details for pharmacovigilance

Prof Thorsten Füreder and Prof Rupert Bartsch
Clinical Division of Oncology
Medical University of Vienna
e-mail: thorsten.fuereder@meduniwien.ac.at
rupert.bartsch@meduniwien.ac.at

Pregnancies

Female of childbearing potentials: Pregnancies and suspected pregnancies (including a positive pregnancy test regardless of age or disease state) of a female subject occurring while the subject is on study drug, or within 28 days of the subject's last dose of study drug, are considered events to be reported immediately to sponsor. If the subject is on study drug, the study drug is to be discontinued immediately and the subject instructed to return any unused portion of the study drug to the investigator. The pregnancy, suspected pregnancy, or positive pregnancy test must be reported to Sponsor. The female should be referred to an obstetrician/gynecologist experienced in reproductive toxicity for further evaluation and counseling.

Male subjects: Female partners of males taking investigational product should be advised to call their healthcare provider immediately if they get pregnant. The male subject should notify the investigator of his partner's pregnancy and her healthcare provider information. The Investigator will then provide this information to the Sponsor and GlaxoSmithKline for follow-up as necessary.

12. QUALITY CONTROL AND QUALITY ASSURANCE

12.1 Periodic monitoring

The designated monitor will contact and visit the investigator regularly and will be allowed to have access to all source documents needed to verify the entries in the case report forms (CRFs) and other protocol-related documents provided that subject confidentiality is maintained in agreement with local regulations. It will be the monitor's responsibility to inspect the CRFs at regular intervals throughout the study, to verify the adherence to the protocol and the completeness, consistency and accuracy of the data being entered on them. The monitoring standards require full verification for the presence of informed consent, adherence to the inclusion/exclusion criteria, documentation of SAEs and the recording of the main efficacy & safety endpoints. To be Good Clinical Practice (GCP) compliant, at least 3 monitoring visits are scheduled. An initiation visit, one routine visit and a final visit after the last patient has finished the study. The monitor will provide a GCP-compliant monitoring report after each visit for the sponsor. The investigator will cooperate with the clinical research associate (CRA) to ensure that any identified discrepancies are resolved

Monitoring will be performed by:

Marika Rosner

Dept of Oncology

Medical University Vienna

12.2 Audits and inspections

The main purpose of an audit or inspection is to confirm that the rights and welfare of the subjects have been adequately protected, and that all data relevant for assessment of safety and efficacy of the investigational product have appropriately been reported. The sponsor/investigator will permit trial related monitoring, audit, institutional review board (IRB) / independent ethic committee (IEC) review & regulatory inspections and provide direct access to study related-source data and source documents.

12.2 Publication and study results

The findings of this study will be published by the Principal Investigator in a scientific journal and presented at scientific meetings.

13. ETHICAL AND LEGAL ASPECTS

13.1 Informed consent of subjects

Following comprehensive instruction regarding the nature, significance, impact and risks of this clinical trial, the patient must give written consent to participation in the study. The patients are made aware of the fact that they can with-draw their consent – without giving reasons – at any time without their further medical care being influenced in any way and they also receive a written patient information sheet in comprehensible language, explaining the nature and purpose of the study and its progress. The patients also must agree to the possibility of study-related data being passed on to relevant authorities. The patients will be informed in detail of their obligations in relation to the insurance in order not to jeopardize insurance cover (also see 13.6).

13.2 Acknowledgment and approval of the study

The investigator submits this protocol and any related document provided to the subject (such as subject information used to obtain informed consent) to an Ethics Committee (EC) or Institutional Review Board (IRB). Approval from the committee must be obtained before starting the study and should be documented in a dated letter to the investigator. Adverse events - whether serious and/or unexpected, and possibly endangering the safety of the study participants - are likewise to be reported to the ethics committee. The clinical trial will be performed in full compliance with the valid legal regulations according to the Drug Law (AMG - Arzneimittelgesetz) of the Republic of Austria. The study will be notified to the Austrian Agency for Health and Food Safety (AGES) and to the European Agency for the Evaluation of Medicinal Products (EMEA) and registered to the European Clinical Trial Database (EudraCT) using the required forms.

13.3 Protocol amendments

Proposed amendments will be submitted to the appropriate competent authorities (CA) and ECs. Substantial amendments may be implemented only after CA/EC approval has been obtained. Amendments that are intended to eliminate an apparent immediate hazard to

subjects will be implemented prior to receiving CA/EC approval. However, in this case, approval must be obtained as soon as possible after implementation.

13.4 Study termination

If the investigator/sponsor decides to terminate the study before it is completed, he will ensure that adequate consideration is given to the protection of the subject interests. The investigator/ sponsor will notify the relevant CA and EC. Documentation will be filed in the trial master and investigator files.

13.5 Clinical study report

Within one year after the final completion of the study, a full clinical study report will be written by the investigator or designee and submitted to the EC and the competent authority. The Principal Investigator will review and sign the final study report.

13.6 Finance and insurance

Finance: Investigator Initiated Trial (ITT)

Insurance: During their participation in the clinical trial the patients will be insured as defined by legal requirements. The sponsor is providing insurance in order to indemnify (legal and financial coverage) the investigator/center against claims arising from the study, except for claims that arise from malpractice and/or negligence. The compensation of the subject in the event of study-related injuries will comply with the applicable regulations.

<u>Details on the existing patients insurance:</u>
Zürich Versicherungs- Aktiengesellschaft; Policy: 07229622-2
This details are also given in the patient information sheet.

13.7 Ethics and good clinical practice

The investigator ensures that this study is conducted in full conformance with the principles of the "Declaration of Helsinki" (as amended at the 56th WMA General Assembly, Tokyo, Japan, 2004), the ICH GCP Guidelines (June 1996) and the laws and regulations of the country in which the clinical research is conducted.

14. REFERENCES

1. Zhu N, Zhang D, Wang W, et al. A Novel Coronavirus from Patients with Pneumonia in China, 2019. *N Engl J Med.* 2020;382(8):727-733.
2. Wu F, Zhao S, Yu B, et al. A new coronavirus associated with human respiratory disease in China. *Nature.* 2020;579(7798):265-269.
3. van Doremalen N, Bushmaker T, Morris DH, et al. Aerosol and Surface Stability of SARS-CoV-2 as Compared with SARS-CoV-1. *N Engl J Med.* 2020.
4. Guan WJ, Ni ZY, Hu Y, et al. Clinical Characteristics of Coronavirus Disease 2019 in China. *N Engl J Med.* 2020.
5. Huang C, Wang Y, Li X, et al. Clinical features of patients infected with 2019 novel coronavirus in Wuhan, China. *Lancet.* 2020;395(10223):497-506.
6. Zhou F, Yu T, Du R, et al. Clinical course and risk factors for mortality of adult inpatients with COVID-19 in Wuhan, China: a retrospective cohort study. *Lancet.* 2020.
7. Wu C, Chen X, Cai Y, et al. Risk Factors Associated With Acute Respiratory Distress Syndrome and Death in Patients With Coronavirus Disease 2019 Pneumonia in Wuhan, China. *JAMA internal medicine.* 2020.
8. Wu Z, McGoogan JM. Characteristics of and Important Lessons From the Coronavirus Disease 2019 (COVID-19) Outbreak in China: Summary of a Report of 72 314 Cases From the Chinese Center for Disease Control and Prevention. *JAMA.* 2020.
9. Cao B, Wang Y, Wen D, et al. A Trial of Lopinavir-Ritonavir in Adults Hospitalized with Severe Covid-19. *N Engl J Med.* 2020.
10. Gautret P, Lagier J-C, Parola P, et al. Hydroxychloroquine and azithromycin as a treatment of COVID-19: results of an open-label non-randomized clinical trial. *International Journal of Antimicrobial Agents.* 2020:105949.
11. Hasibeder W KM, Müller-Muttonen St, Markstaller K, Likar R. OEGARI Empfehlung COVID 19 (Österreichische Gesellschaft für Anästhesiologie, Reanimation und Intensivmedizin). 2020.
12. https://www.who.int/emergencies/diseases/novel-coronavirus-2019.
13. Liang W, Guan W, Chen R, et al. Cancer patients in SARS-CoV-2 infection: a nationwide analysis in China. *Lancet Oncol.* 2020;21(3):335-337.
14. https://www.onkopedia.com/de/onkopedia/guidelines. *Leitlinie: Coronavirus-Infektion (COVID-19) bei Patienten mit Blut- und Krebserkrankungen (Stand 25032020).*
15. Van Nuffel AM, Sukhatme V, Pantziarka P, Meheus L, Sukhatme VP, Bouche G. Repurposing Drugs in Oncology (ReDO)-clarithromycin as an anti-cancer agent. *Ecancermedicalscience.* 2015;9:513.
16. Cramer CL, Patterson A, Alchakaki A, Soubani AO. Immunomodulatory indications of azithromycin in respiratory disease: a concise review for the clinician. *Postgraduate medicine.* 2017;129(5):493-499.
17. Ferreri AJM, Cecchetti C, Kiesewetter B, et al. Clarithromycin as a "repurposing drug" against MALT lymphoma. *Br J Haematol.* 2018;182(6):913-915.
18. Zimmermann P, Ziesenitz VC, Curtis N, Ritz N. The Immunomodulatory Effects of Macrolides-A Systematic Review of the Underlying Mechanisms. *Frontiers in immunology.* 2018;9:302.
19. Ratzinger F, Haslacher H, Poeppl W, et al. Azithromycin suppresses CD4+ T-cell activation by direct modulation of mTOR activity. *Scientific Reports.* 2014;4(1):7438.
20. Ferreri AJ, Sassone M, Kiesewetter B, et al. High-dose clarithromycin is an active monotherapy for patients with relapsed/refractory extranodal marginal zone lymphoma of mucosa-associated lymphoid tissue (MALT): the HD-K phase II trial. *Ann Oncol.* 2015;26(8):1760-1765.
21. Govi S, Dognini GP, Licata G, et al. Six-month oral clarithromycin regimen is safe and active in extranodal marginal zone B-cell lymphomas: final results of a single-centre phase II trial. *Br J Haematol.* 2010;150(2):226-229.
22. Lagler H, Kiesewetter B, Dolak W, et al. Treatment of mucosa associated lymphoid tissue lymphoma with a long-term once-weekly regimen of oral azithromycin: Results from the phase II MALT-A trial. *Hematol Oncol.* 2019;37(1):22-26.
23. Ling KM, Hillas J, Lavender MA, et al. Azithromycin reduces airway inflammation induced by human rhinovirus in lung allograft recipients. *Respirology (Carlton, Vic).* 2019;24(12):1212-1219.
24. Bauer P, Bretz F, Dragalin V, König F, Wassmer G. Twenty-five years of confirmatory adaptive designs: opportunities and pitfalls. *Statistics in medicine.* 2016;35(3):325-347.
25. Bretz F, Koenig F, Brannath W, Glimm E, Posch M. Adaptive designs for confirmatory clinical trials. *Statistics in medicine.* 2009;28(8):1181-1217.
26. Lehmacher W, Wassmer G. Adaptive sample size calculations in group sequential trials. *Biometrics.* 1999;55(4):1286-1290..
