# Supplementary material for: Prophylactic treatment with oral azithromycin in cancer patients during the COVID-19 pandemic (OnCoVID): a randomized, single-blinded, placebo-controlled phase 2 trial
Source: Infect Agent Cancer. 2023 Feb 12;18:9. doi: 10.1186/s13027-023-00487-x (PMC9924847; doi:10.1186/s13027-023-00487-x)
Supplement: Supplementary file 2 — Additional file 2: Table S1. Minimal Inhibitory Concentrations (MIC) of azithromycin resistance testing for individual patients in the placebo and azithromycin groups. Table S2. Descriptive data for inflammatory parameters of the included patients. Tables S3–S10. Generalized mixed model parameters for inflammatory parameters. [file 13027_2023_487_MOESM2_ESM.pdf]

Supplement B

Supplementary Table 1. Minimal Inhibitory Concentrations (MIC) for individual patients in the placebo and azithromycin groups. MIC values were evaluated in triplicates and are given in mg/L.

	**Placebo (n = 5)**	**Azithromycin (n = 4)**
**Visit**	**Median MIC in mg/L**	**Median MIC in mg/L**
Baseline	0.5	0.5
Visit 1	0.5	0.5
Visit 2	0.5	0.5
Baseline	32	32
Visit 1	32	32
Visit 2	32	32
Baseline	1	32
Visit 1	1	32
Visit 2	1	32
Baseline	1	32
Visit 1	1	32
Visit 2	1	32
Baseline	1	
Visit 1	1	
Visit 2	1	

Supplementary Table 2. Median/range of inflammatory markers in the azithromycin and placebo groups at each of the three visits.

Character-istics	Azithromycin						Placebo					
	Visit 1 Median (Range)		Visit 2 Median (Range)		End of study Median (Range)		Visit 1 Median (Range)		Visit 2 Median (Range)		End of study Median (Range)	
NLR	2.95	0.08; 12.91	2.55	0.63; 20.68	3.05	1.00; 18.57	4.38	1.35; 25.00	3.03	0.82; 20.40	2.33	1; 15.25
LLR	4.71	1.2; 14.34	4.26	1.89; 25.16	4.84	0.03; 33.21	5.86	2.86; 27.57	4.77	2.23; 22.36	4.06	0.03; 18.73
PLR	176.9	7.3; 597.1	206.5	39.8; 787.8	159.3	1.7; 875.6	283.6	71.4; 1586.7	211.7	90.2; 1138.8	149.9	1.18; 566.7
MLR	0.40	0.02; 5.62	0.50	0.13; 1.53	0.43	0.13; 6.53	0.43	0.00; 4.61	0.51	0.04; 1.81	0.41	0.17; 2.69
CRP	0.35	0.03; 5.78	0.36	0.02; 9.67	0.46	0.05; 17.29	0.54	0.03; 10.14	0.41	0.03; 20.30	0.34	0.03; 3.17
CRP/Alb	0.90	0.07; 15.64	0.96	0.07; 26.79	1.24	0.12; 75.5	1.32	0.07; 29.31	1.16	0.07; 63.24	0.86	0.08; 9.03
PCT	0.05	0.02; 13.99	0.05	0.02; 99.00	0.06	0.02; 1.65	0.04	0.02; 14.00	0.04	0.02; 99.00	0.04	0.02; 15.8
IL-6	4.19	1.50; 48.42	5.92	1.50; 60.57	4.38	1.50; 32.85	2.95	0.33; 99.00	4.24	1.5; 31.10	4.17	1.5; 21.77

CRP: C-reactive protein, NLR: neutrophil/lymphocyte ratio, LLR: leukocyte/lymphocyte ratio, PLR: platelet/lymphocyte ratio, MLR: monocyte/lymphocyte ratio, CRP/Alb: C-reactive protein/albumin ratio, IL-6: Interleukin-6, PCT: Procalcitonin

Supplementary Table 3. Generalized linear mixed model predicting the neutrophil/lymphocyte ratio (NLR). Likelihood ratio test (LRT) for interaction between treatment group and visit: p = 0.008.

**Parameter**	**Coefficient**	**SE**	**p-value**
Intercept	1.66	0.163	
Verum	-0.60	0.230	0.009
Follow-up 1	-0.51	0.165	0.002
Follow-up 2	-0.64	0.175	<0.001
Verum x Follow-up 1	0.41	0.232	0.077
Verum x Follow-up 2	0.76	0.244	0.002

Supplementary Table 4. Generalized linear mixed model predicting the leukocyte/lymphocyte ratio (LLR). Likelihood ratio test (LRT) for interaction between treatment group and visit: p = 0.004.

**Parameter**	**Coefficient**	**SE**	**p-value**
Intercept	2.02	0.109	
Verum	-0.43	0.154	0.005
Follow-up 1	-0.36	0.102	<0.001
Follow-up 2	-0.47	0.123	<0.001
Verum x Follow-up 1	0.33	0.144	0.024
Verum x Follow-up 2	0.57	0.171	0.001

Supplementary Table 5. Generalized linear mixed model predicting the platelet/lymphocyte ratio (PLR). Likelihood ratio test (LRT) for interaction between treatment group and visit: p = 0.116.

**Parameter**	**Coefficient**	**SE**	**p-value**
Intercept	5.54	0.117	
Verum	-0.23	0.132	0.082
Follow-up 1	-0.11	0.089	0.222
Follow-up 2	-0.26	0.114	0.020

Supplementary Table 6. Generalized linear mixed model predicting the monocyte/lymphocyte ratio (MLR). Likelihood ratio test (LRT) for interaction between treatment group and visit: p = 0.621.

**Parameter**	**Coefficient**	**SE**	**p-value**
Intercept	-0.61	0.109	
Verum	0.01	0.119	0.946
Follow-up 1	0.03	0.097	0.735
Follow-up 2	0.10	0.099	0.333

Supplementary Table 7. Generalized linear mixed model predicting C-reactive protein (CRP) levels. Likelihood ratio test (LRT) for interaction between treatment group and visit: p = 0.173.

**Parameter**	**Coefficient**	**SE**	**p-value**
Intercept	-0.35	0.206	
Verum	-0.02	0.268	0.933
Follow-up 1	-0.07	0.105	0.523
Follow-up 2	-0.07	0.143	0.642

Supplementary Table 8. Generalized linear mixed model predicting the CRP/albumin ratio (CRP/alb). Likelihood ratio test (LRT) for interaction between treatment group and visit: p = 0.097.

**Parameter**	**Coefficient**	**SE**	**p-value**
Intercept	0.23	0.274	
Verum	-0.04	0.356	0.904
Follow-up 1	0.01	0.155	0.926
Follow-up 2	-0.01	0.188	0.954

Supplementary Table 9. Generalized linear mixed model predicting procalcitonin (PCT) levels. Likelihood ratio test (LRT) for interaction between treatment group and visit: p = 0.234.

**Parameter**	**Coefficient**	**SE**	**p-value**
Intercept	-1.51	0.162	
Verum	-0.16	0.193	0.411
Follow-up 1	-0.08	0.093	0.409
Follow-up 2	-0.03	0.095	0.770

Supplementary Table 10. Generalized linear mixed model predicting interleukin-6 (IL-6) levels. Likelihood ratio test (LRT) for interaction between treatment group and visit: p = 0.729.

**Parameter**	**Coefficient**	**SE**	**p-value**
Intercept	1.34	0.174	
Verum	0.19	0.222	0.387
Follow-up 1	0.20	0.104	0.053
Follow-up 2	0.18	0.124	0.148
